# Physicochemical and Functional Properties of Yanbian Cattle Bone Gelatin Extracted Using Acid, Alkaline, and Enzymatic Hydrolysis Methods

**DOI:** 10.3390/gels11030186

**Published:** 2025-03-07

**Authors:** Song Zhang, Duanduan Zhao, Lu Yin, Ruixuan Wang, Zhiyan Jin, Hongyan Xu, Guangjun Xia

**Affiliations:** 1 Department of Food Science and Engineering, College of Agriculture, Yanbian University, Yanji 133000, China; 2022010679@ybu.edu.cn (S.Z.); 18943362092@163.com (D.Z.); 18743675027@163.com (L.Y.); 2024010885@ybu.edu.cn (R.W.); 15526722180@163.com (Z.J.); 2 Department of Animal Science, College of Agriculture, Yanbian University, Yanji 133000, China

**Keywords:** Yanbian cattle, bone, gelatin, enzymatic hydrolysis, physicochemical properties

## Abstract

Yanbian cattle, a high-quality indigenous breed in China, were selected due to their unique biological characteristics, underutilized bone byproducts, and potential as a halal-compliant gelatin source, addressing the growing demand for alternatives to conventional mammalian gelatin in Muslim-majority regions. This study investigates the physicochemical and functional properties of gelatin extracted from Yanbian cattle bones using three different methods: acid, alkaline, and papain enzymatic hydrolysis. The extraction yields and quality of gelatin were evaluated based on hydroxyproline content, gel strength, viscosity, amino acid composition, molecular weight distribution, and structural integrity. Specifically, A gelatin, prepared using 0.075 mol/L hydrochloric acid, achieved the highest yield (18.64%) among the acid-extraction methods. B gelatin, extracted with 0.1 mol/L sodium hydroxide, achieved the highest yield (21.06%) among the alkaline-extraction methods. E gelatin, obtained through papain hydrolysis, exhibited the highest yield (25.25%) among the enzymatic methods. Gelatin extracted via papain enzymatic hydrolysis not only retained better protein structure but also exhibited higher hydroxyproline content (19.13 g/100 g), gel strength (259 g), viscosity (521.67 cP), and superior thermal stability. Structural analyses conducted using SDS-PAGE, GPC, FTIR, XRD, and CD spectroscopy confirmed that papain extraction more effectively preserved the natural structure of collagen. Furthermore, amino acid composition analysis revealed that gelatin extracted via papain hydrolysis contained higher levels of essential residues, such as glycine, proline, and hydroxyproline, emphasizing the mild and efficient nature of enzymatic treatment. These findings suggest that, compared with acid and alkaline extraction methods, enzymatic hydrolysis has potential advantages in gelatin production. Yanbian cattle bone gelatin shows promise as an alternative source for halal gelatin production. This study also provides insights into optimizing gelatin production to enhance its functionality and sustainability.

## 1. Introduction

Gelatin, a biopolymer derived from the partial hydrolysis of collagen, is widely utilized in the food, pharmaceutical, and biomedical industries due to its biocompatibility, biodegradability, and excellent film-forming and gelling properties [1,2]. For example, a recent study explored the influence of gelatin/guar gum mixtures on the rheological and textural properties of restructured ricotta cheese. The combination of gelatin and guar gum significantly improved the texture and viscosity of the cheese, making it a promising candidate for food products requiring improved mouthfeel and stability [3]. Zhao et al. used citric acid as a cross-linking agent to form a film matrix through the condensation reaction between bovine bone gelatin and chitosan. Using the blueberry anthocyanin–iron ion complex as a pH-sensitive indicator, they developed a highly sensitive indicator film to effectively monitor the freshness of pork [4]. Traditional gelatin extraction from mammalian sources (e.g., bovine bones) faces limitations in meeting halal certification requirements, driving demand for alternative processing methods and underutilized raw materials [5]. Yanbian cattle, a high-quality Chinese breed with unique collagen composition due to genetic and environmental factors, offer a promising yet underexplored gelatin source [6]. Comparative studies on extraction methods for this specific collagen source remain scarce, particularly regarding structural preservation and functional performance.

Currently, three main methods are used for gelatin production: the alkaline method, the acid method, and the enzymatic method. Ismail and Abdullah treated fish skin with hydrochloric acid (HCl) at different concentrations (0.05–0.2 M), followed by thermal extraction of gelatin in a water bath at 45 °C. The results showed that the gelatin yields were 18.86% and 20.95% when using 0.05 M and 0.2 M HCl, respectively [7]. Similarly, Amertaningtyas et al. compared acid (0.25 M HCl) and alkaline (0.25 M NaOH) pretreatments for cattle hide-derived gelatin, revealing that HCl pretreatment yielded superior outcomes in terms of extraction efficiency, protein content, and gel strength. Alkaline methods, while advantageous for mature collagen sources (e.g., cattle bones and ligaments) due to their ability to preserve cross-linked structures and minimize yield loss, may inadvertently reduce yields if excessively prolonged, as prolonged alkali exposure increases collagen solubility in aqueous phases [8,9]. For instance, Mafazah et al. identified 0.01 M NaOH with a 12 h treatment as optimal for fish skin gelatin extraction, noting that alkaline conditions induce deamidation of glutamine and asparagine residues, shifting the isoelectric point from 7–9 to 5 [10,11]. However, alkaline-derived gelatin typically exhibits lower yields and higher lipid residues compared to acid-based methods [12]. In contrast, enzymatic strategies utilizing proteases (e.g., papain) offer a sustainable alternative by hydrolyzing collagen from animal byproducts into gelatin with enhanced efficiency. This biocatalytic approach not only shortens processing times but also improves yield and reduces waste generation [13]. Ma et al. selected a protease with specific catalytic specificity, pepsin, which remains stable and effectively cleaves peptide bonds under low pH conditions. They then innovatively combined the demineralization process (using hydrochloric acid to dissolve hydroxyapatite in bones) with pepsin hydrolysis in a single-step reaction. By eliminating the lime treatment and demineralization steps found in traditional methods, this approach reduces environmental pollution. As a result, a high-quality gelatin product with improved physicochemical properties was obtained compared to the gelatin extracted by traditional methods [14]. Zaitsev et al. have demonstrated that the molecular weight distribution (MWD) of gelatin significantly influences its properties [15]. To preserve the structural integrity of collagen, it is crucial to select enzymes that cleave collagen chains gently while minimizing damage to critical structural domains. Enzymes such as papain exhibit advantages in this regard, as they specifically target and cleave certain peptide bonds rather than randomly disrupting the three-dimensional structure of collagen. This selective cleavage can help maintain or enhance the content of key amino acids such as hydroxyproline, which plays a vital role in determining the properties of gelatin [16,17]. However, comparative studies on extraction methods for specific collagen sources remain scarce, particularly regarding structural preservation and functional performance.

This study aimed to comprehensively evaluate and compare the properties of gelatin extracted from Yanbian cattle bone using acid, alkaline, and enzymatic hydrolysis. Specifically, the study focused on two primary objectives: (1) Structural analysis: Investigating the molecular structural characteristics and molecular weight distribution of gelatin using spectroscopic and chromatographic techniques to understand the degree of collagen degradation and preservation of the triple-helix structure in each extraction method. (2) Functional property assessment: Examining the functional properties of gelatin, including moisture content, crude protein content, ash content, gel strength, and viscosity, which are critical for its application in the food industry. The findings provide valuable insights into how different extraction methods affect the characteristics and performance of Yanbian cattle bone gelatin, thereby contributing to the development of high-value-added products from sustainable and underutilized animal byproducts.

## 2. Results and Discussion

### 2.1. Yield of Extraction

**Figure 1:**
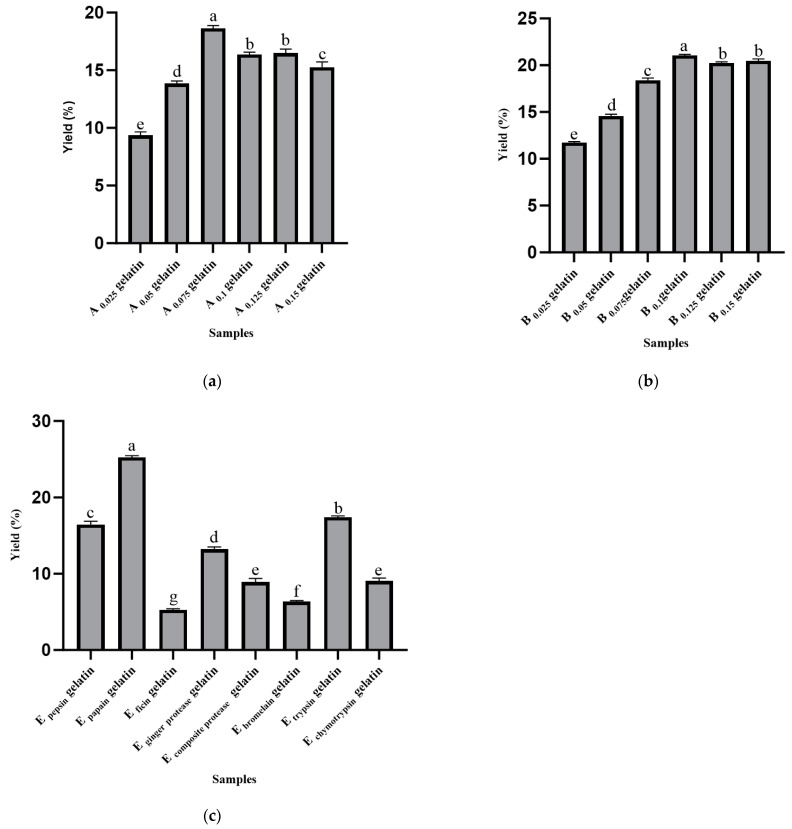
Yields (%) extracted from Yanbian cattle bone using different methods. (**a**) A_0.025_ gelatin, A_0.05_ gelatin, A_0.075_ gelatin, A_0.1_ gelatin, A_0.125_ gelatin, and A_0.15_ gelatin denote the gelatins extracted with different hydrochloric acid concentrations. (**b**) B_0.025_ gelatin, B_0.05_ gelatin, B_0.075_ gelatin, B_0.1_ gelatin, B_0.125_ gelatin, and B_0.15_ gelatin denote the gelatins extracted with different sodium hydroxide concentrations. (**c**) E _pepsin_ gelatin, E _papain_ gelatin, E _ficin_ gelatin, E _ginger protease_ gelatin, E _composite protease_ gelatin, E _bromelain_ gelatin, E _trypsin_ gelatin, and E _chymotrypsin_ gelatin denote the gelatins extracted with different enzymes. Results are presented as mean ± SD (*n* = 3). Statistical analysis was performed between samples for the same property. Different superscript letters (a–g) within the same color of bar chart indicate statistically significant differences as determined by one-way ANOVA followed by Duncan’s post hoc test (*p* < 0.05).

Figure 1a shows the effect of different hydrochloric acid concentrations on gelatin yield. The yield of A 0.075 gelatin was significantly higher than that of other concentration groups (*p* < 0.05). As the concentration of hydrochloric acid increased, the yield initially increased, reaching its maximum at 0.075 mol/L, and then decreased. This phenomenon may be attributed to the fact that an appropriate acid concentration can effectively break cross-linked structures, producing more gelatin, while excessively high concentrations may lead to excessive degradation of collagen, thereby reducing the yield. A certain concentration of acid disrupts the cross-linking between collagen fibers, leading to the degradation of collagen chains into smaller peptides. This degradation not only reduces the molecular weight of collagen but also results in the loss of functional amino acids, such as hydroxyproline, which are essential for maintaining the structural integrity of collagen and the yield of gelatin [18]. Figure 1b shows the effect of different sodium hydroxide concentrations on gelatin yield. The yield of B 0.1 gelatin was significantly higher than that of other samples (*p* < 0.05). The results indicate that an increase in sodium hydroxide concentration facilitates the removal of non-collagenous proteins and lipids, thereby promoting the release of gelatin. However, when the concentration exceeded 0.1 mol/L, the yield slightly decreased, which may be attributed to the strong corrosiveness of the alkali causing excessive structural damage and affecting the extraction efficiency. Compared to acid treatment, the hydrolysis process of collagen under lower concentrations of NaOH is relatively milder. While alkaline conditions can also degrade the collagen structure, the effect is slower, allowing for better preservation of the collagen’s molecular structure. Hydroxide ions (OH-) primarily act on non-collagenous proteins and lipids, rather than directly attacking the collagen backbone, thus the hydrolysis process is relatively slower, and the degree of degradation is milder. Under lower concentrations of sodium hydroxide, the alkaline hydrolysis generally occurs at a slower and less pronounced rate than acidic hydrolysis. This difference is supported by findings in the literature, such as Gómez-Guillén et al., who reported that acid treatments at higher concentrations lead to a more extensive breakdown of collagen’s molecular structure compared to alkaline treatments [19]. Additionally, Cao et al. also observed that acid pretreatment causes more severe molecular weight reduction in collagen, whereas alkaline treatment preserves the collagen structure to a greater extent [20]. Figure 1c illustrates the effect of different enzyme treatments on gelatin yield. E papain gelatin showed a significantly higher yield compared to other enzyme-treated samples (*p* < 0.05). The catalytic mechanism of papain is determined by the cysteine-25 triad located at its active site, which operates effectively when the pH is maintained between 3.0 and 9.0 and the temperature ranges from 60 to 70 °C. During this process, asparagine-175 and histidine-159 work synergistically to cleave peptide bonds by attracting the carbonyl carbon of the peptide backbone, thereby releasing the amino-terminal end [16]. This explains the strong catalytic activity of papain, making it suitable for extracting bone gelatin. The high degree of cross-linking within collagen molecules is primarily determined by the presence of certain amino acids, such as proline and hydroxyproline, which are essential for the formation and stabilization of the collagen triple helix. The cross-linking is further influenced by environmental factors such as pH, temperature, and the concentration of specific ions or chemicals used during extraction. In particular, acidic or alkaline conditions can disrupt these cross-links, causing either partial or complete degradation of the collagen structure. The degree of cross-linking in collagen is essential for its functional properties, such as gel strength, viscosity, and thermal stability [21]. On the other hand, enzymatic hydrolysis using proteases offers a milder approach that specifically targets collagen without causing excessive degradation, thus enabling the more efficient release of high-quality gelatin. Therefore, the release conditions, such as pH, enzyme concentration, and reaction time, need to be carefully controlled to achieve an optimal balance between cross-link disruption and the preservation of collagen structure, leading to high yields and high-quality gelatin [22].

### 2.2. Physicochemical Properties

#### 2.2.1. Moisture Content

As shown in Table 1, moisture content was consistent across the three methods: enzymatic, alkaline, and acid. This similarity suggests that the final drying processes were effectively controlled and that the extraction method had minimal impact on moisture retention in the gelatin samples. The lack of significant differences in moisture content across the different extraction methods (acid, alkaline, and enzymatic) can be attributed to the effectiveness of the final drying process in all methods. After the gelatin was extracted, it underwent a similar drying procedure, which controlled the moisture content and likely minimized the impact of the extraction method on the final moisture levels. Moisture content in gelatin is primarily influenced by the drying conditions rather than the extraction process itself [23]. In gelatin production, stable moisture content is important, as it affects the shelf life, handling, and susceptibility to microbial growth [24]. Since there were no significant differences in moisture content among the methods, this parameter does not distinguish product quality. Therefore, the primary factors influencing gelatin quality are more likely related to protein integrity and yield rather than moisture content.

#### 2.2.2. Ash Content

Ash content, an important indicator of gelatin quality, reflects the residual inorganic matter in the product. As shown in Table 1, the ash content of gelatin obtained from different extraction methods showed no significant differences. These ash values indicated that the three methods were similarly effective in removing inorganic substances. Ash content is closely related to the purity of gelatin, with lower ash contents indicating higher purity, which is particularly important for applications in food and pharmaceuticals. The low ash content observed in all gelatin samples suggests high purity, making these gelatins suitable for applications requiring high quality [25]. Furthermore, low ash content contributes to an improved transparency and sensory quality of gelatin, preventing color darkening and enhancing transparency. It also helps to maintain desirable gel strength and solubility.

#### 2.2.3. Crude Protein Content

As shown in Table 1, enzymatic extraction yielded the highest crude protein content, followed by alkaline and the acid methods. A higher protein content typically indicates a greater proportion of collagen in the final product, as impurities and non-collagen proteins are minimized [26]. The reason why enzymatic extraction results in a higher crude protein content is that during enzymatic hydrolysis, the enzyme specifically targets collagen molecules, selectively cleaving peptide bonds and avoiding excessive degradation of the collagen. This gentle treatment preserves more collagen molecules, resulting in a higher proportion of collagen in the final extracted gelatin. Therefore, enzymatic extraction better preserves the structure and protein content of collagen. In contrast, acid or alkaline extraction methods may cause partial degradation of collagen, leading to a reduction in protein content [15]. The selective cleavage of papain likely reduces non-collagen protein contaminants, thereby enhancing the purity and functionality [27]. In contrast, acid extraction may partially hydrolyze the collagen backbone, resulting in reduced protein content and molecular breakdown [28]. The high protein content of the enzymatically extracted gelatin is particularly valuable for applications requiring high gel strength and purity.

#### 2.2.4. Hydroxyproline Content

As shown in Table 1, hydroxyproline content, a key marker of collagen integrity, was highest in enzymatically extracted gelatin, followed by the alkaline and the acid methods. The higher hydroxyproline content in enzymatic gelatin reflects better preservation of the triple-helical structure of collagen, as hydroxyproline stabilizes collagen fibers through hydrogen bonding and supports gel strength [29]. The selective action of papain likely prevents the extensive molecular breakdown observed with hydrochloric acid and sodium hydroxide treatments, which can cause excessive denaturation or decarboxylation of hydroxyproline residues.

In acidic environments, hydrogen ions (H+) can promote the removal of a hydrogen ion from the carboxyl group (-COOH) of hydroxyproline residues, forming a carboxylate salt, which may lead to a decarboxylation reaction. Under stronger acidic conditions, acids not only protonate functional groups such as amino and carboxyl groups but may also cause collagen degradation. The decarboxylation of hydroxyproline alters its chemical structure, thereby affecting the stability of collagen. Excessive acidic treatment can lead to the decarboxylation of hydroxyproline, which in turn reduces the quality of gelatin, particularly affecting its structural stability. In alkaline conditions, hydroxide ions (OH-) can deprotonate the carboxyl group, causing it to enter a deprotonated state. This process can disrupt the normal structure of hydroxyproline, leading to the loss of a hydrogen ion and subsequent decarboxylation. Deprotonation in alkaline environments may not only result in the decarboxylation of hydroxyproline but also further disrupt the collagen triple-helix structure, affecting the stability of collagen and the functionality of gelatin [30]. In contrast to these two treatments, papain, as a cysteine protease, exhibits strong selectivity. It specifically cleaves peptide bonds within collagen, primarily targeting non-collagenous components while having a minimal impact on the core collagen structure, thereby preserving the integrity of collagen. This selective action ensures that the hydroxyproline content remains higher, leading to improved gelatin quality [31]. This structural preservation enhances the functional properties of gelatin, particularly gel strength, elasticity, and thermal stability—critical for high-value applications such as pharmaceuticals, biomedical materials, and high-quality food gels [32].

#### 2.2.5. Gel Strength

Gelatin gel strength is a critical parameter reflecting the structural integrity and functionality of extracted collagen. As shown in Table 2, E gelatin, prepared using the enzymatic method, exhibited the highest gel strength, significantly outperforming B gelatin from the alkaline method and A gelatin from the acid method. The superior gel strength of E gelatin indicates that the papain process preserves the triple-helical structure of collagen more effectively. The gentle and specific action of papain minimizes the breakdown of collagen chains compared to the more aggressive hydrolysis observed with hydrochloric acid and sodium hydroxide treatments. The triple-helical structure of collagen is formed by three tightly intertwined polypeptide chains (α-chains) stabilized through hydrogen bonds and van der Waals forces. The non-helical telopeptide regions at the termini of collagen molecules serve as critical sites for intermolecular cross-linking. Papain, a cysteine protease with substrate specificity, preferentially hydrolyzes the non-helical telopeptide regions of collagen molecules, which are characterized by loose conformations or specific amino acid sequences, while avoiding direct cleavage of the triple-helical core domain. This selective cleavage releases intact helical domains, thereby preserving the integrity of the triple-helical structure. The triple-helical regions, enriched with glycine, proline, and hydroxyproline, exhibit strong resistance to papain-mediated proteolysis due to their rigid conformation conferred by the closely packed arrangement of these amino acids [33]. Consequently, the core triple-helical structure remains undamaged. The retention of relatively intact triple-helical fragments enables the formation of a denser three-dimensional network during gelation through hydrogen bonding and hydrophobic interactions. Furthermore, the residual triple-helical structures act as “physical cross-linking junctions”, enhancing intermolecular interactions within the gelatin matrix and ultimately improving gel strength. This makes E gelatin ideal for applications requiring robust gel formation, such as high-quality gelatin desserts, gummy candies, and pharmaceutical gel capsules, where mechanical strength and texture are essential [34].

#### 2.2.6. Viscosity

Viscosity plays a crucial role in determining the texture and stability of gelatin solutions, thereby influencing their suitability as thickening agents in various food and pharmaceutical products [35]. As shown in Table 2, the papain-extracted gelatin (E gelatin) also showed the highest viscosity, compared to the acid and alkaline extractions. The high gel strength and high viscosity of E gelatin are interconnected, as both properties rely on the structural integrity of collagen molecules [24]. The triple-helix structure of collagen plays a crucial role in forming a strong gel network. E gelatin retains a higher content of hydroxyproline, effectively maintaining the collagen triple-helix structure, which allows the gelatin to form a stronger gel network upon cooling, thereby enhancing gel strength. At the same time, E gelatin better preserves the integrity of the collagen structure, resulting in a higher molecular weight, which increases its water-holding capacity and thus improves its viscosity. This means that larger and more intact collagen molecules enable the gelatin to form a more stable gel, increasing the viscosity of the solution, and ultimately resulting in higher gel strength and viscosity.

#### 2.2.7. pH

As shown in Table 2, A gelatin exhibits significant differences in pH compared to B gelatin and E gelatin. This indicates that gelatin produced using the acidic method retains a certain level of acidity, it is likely due to the use of hydrochloric acid during the extraction process. During this process, some of the acid may remain in the final gelatin product, causing it to be acidic [36]. whereas gelatin produced using the alkaline method shows a neutral pH, and the enzymatic method yields a slightly acidic pH. The near-neutral to slightly acidic pH value of the enzymatic method may help maintain the natural properties of gelatin and improve its stability in certain food systems. The higher pH value of the alkaline method may be disadvantageous for food applications sensitive to pH changes, whereas the lower pH of acidic gelatin may limit its use in certain neutral food systems. However, acidic gelatin could be advantageous for acidic food formulations, as a neutral pH minimizes undesirable interactions with other food systems, ensuring consistent texture and taste [37]. Additionally, compared to gelatin A, gelatin B and gelatin E are more suitable for incorporation into food systems. The higher pH value of the alkaline method may be disadvantageous for food applications sensitive to pH changes, whereas the lower pH of acidic gelatin may limit its use in certain neutral food systems. However, acidic gelatin could be advantageous for acidic food formulations, as a neutral pH minimizes undesirable interactions with other food systems, ensuring consistent texture and taste [37].

#### 2.2.8. Isoelectric Point

The isoelectric point represents the pH at which gelatin carries no net charge. As shown in Table 2. The high isoelectric point of E gelatin may result from the selective hydrolysis of specific amino acid residues by papain, yielding a structure closer to its native state. Differences in the isoelectric points directly affect the solubility and stability of gelatin under different pH conditions. The high isoelectric point of E gelatin suggests better solubility and thermal stability in neutral and alkaline conditions. In contrast, the lower isoelectric point of B gelatin may render it more prone to precipitation in acidic environments, potentially affecting its dispersion and emulsification properties in some food applications [38].

### 2.3. Rheological Properties of Gelatin

**Figure 2:**
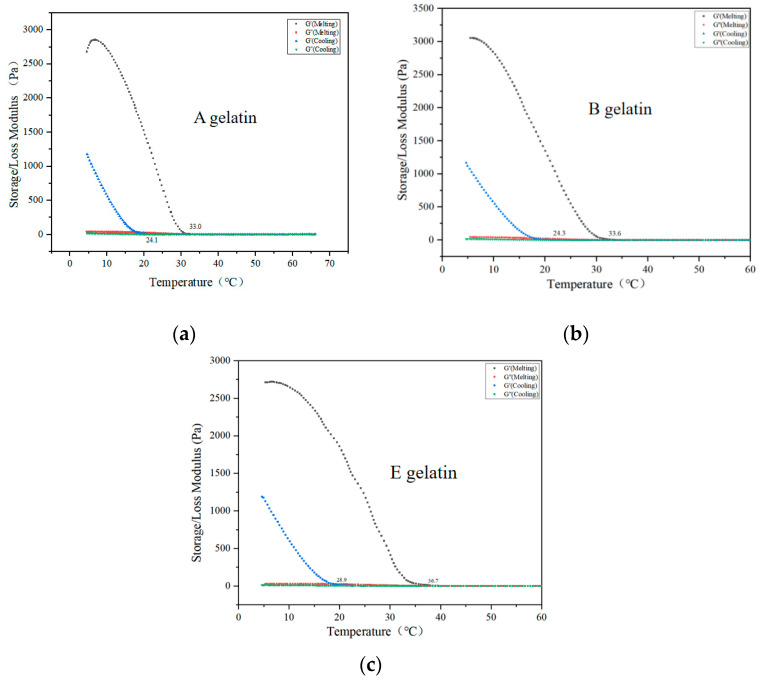
Viscoelastic properties (**a**–**c**) of A gelatin, B gelatin, and E gelatin. A gelatin denotes the gelatins extracted with 0.075 mol/L hydrochloric acid. B gelatin denotes the gelatins extracted with 0.1 mol/L sodium hydroxide. E gelatins denote the gelatins extracted with papain. hey are the highest-yielding samples from the three methods (acid method, alkaline method, and enzymatic method), respectively.

The gelation and melting temperatures are key parameters for evaluating the gelling characteristics of gelatin [20]. Figure 2 shows the dynamic viscoelastic curves of A, B, and E gelatins during the cooling and heating processes. The storage modulus (G′) and loss modulus (G′) intersect, defining the gelation and melting temperatures, respectively [39]. Figure 2a shows the rheological behavior of A gelatin. During cooling, the G′ (storage modulus) of A gelatin significantly increases at around 24.1 °C, indicating the onset of gelation. As the temperature decreases further, the G′ value rises rapidly, reflecting the progressive formation and stabilization of the gelatin network. During the cooling process of A gelatin, a phase transition occurs from a liquid to a gel state. This process is driven by molecular interactions between collagen molecules, particularly hydrogen bonding, which promotes the aggregation and network formation of gelatin molecules. As the gel structure gradually forms, the G′ value increases [40]. The triple-helix structure of collagen may be partially disrupted during the extraction process, but as the temperature decreases, the triple-helix structure begins to reassemble and stabilize, strengthening the gel network and causing a significant increase in G′ [41]. Conversely, G″ (loss modulus) decreases at lower temperatures, indicating the dominance of elasticity in the gelatin structure. A gelatin exhibits a melting temperature of approximately 33.0 °C, indicating relatively low thermal stability. This suggests that acid-extracted gelatin forms elastic gels at lower temperatures, making it suitable for applications requiring quick setting and low melting points. Figure 2b shows the rheological characteristics of gelatin B. Compared to A gelatin, B gelatin has a slightly higher gelation point (24.3 °C) and a melting temperature (33.6 °C). The G′ and G″ modulus curves were relatively smooth, indicating that alkaline treatment had a milder impact on the collagen structure. This results in stable rheological properties over a broader temperature range. Figure 2c shows the rheological analysis of gelatin E. Compared to A and B gelatins, E gelatin exhibits a significantly higher gelation point (approximately 28.9 °C) and the highest melting temperature (36.7 °C). The enzymatic treatment with papain significantly enhances the thermal stability of the gelatin. Additionally, the G′ modulus of E gelatin remains higher than that of A and B samples across all temperature ranges, demonstrating superior gel-forming capability and structural stability. Ma et al. and Sarbon et al. reported that the intrinsic differences in the gelatin structure and the processing methods used in gelatin production can lead to variations in gelling ability [14,36]. Abedinia et al. also reported that the source of gelatin, breed and age of the animal, molecular weight, peptide chain cleaving position, and the concentration of amino acid residue in gelatin are the key contributors to the rheological properties of gelatin [36]. This is consistent with the conclusions of this study.

### 2.4. The Protein Structure of Gelatin

#### 2.4.1. SDS-PAGE

**Figure 3:**
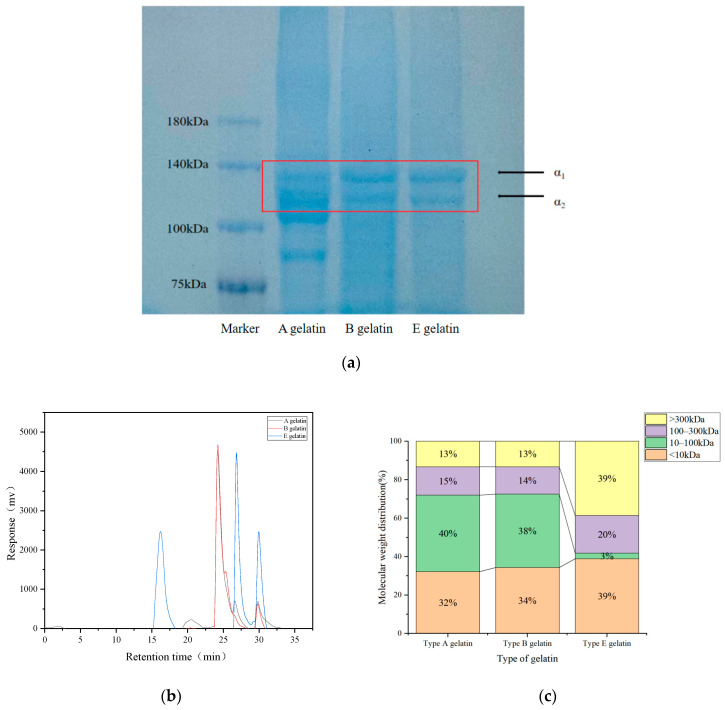
SDS-PAGE patterns: (**a**) molecular weight distribution and (**b**) molecular weight ratio (**c**) of A gelatin, B gelatin, and E gelatin.

**Figure 4:**
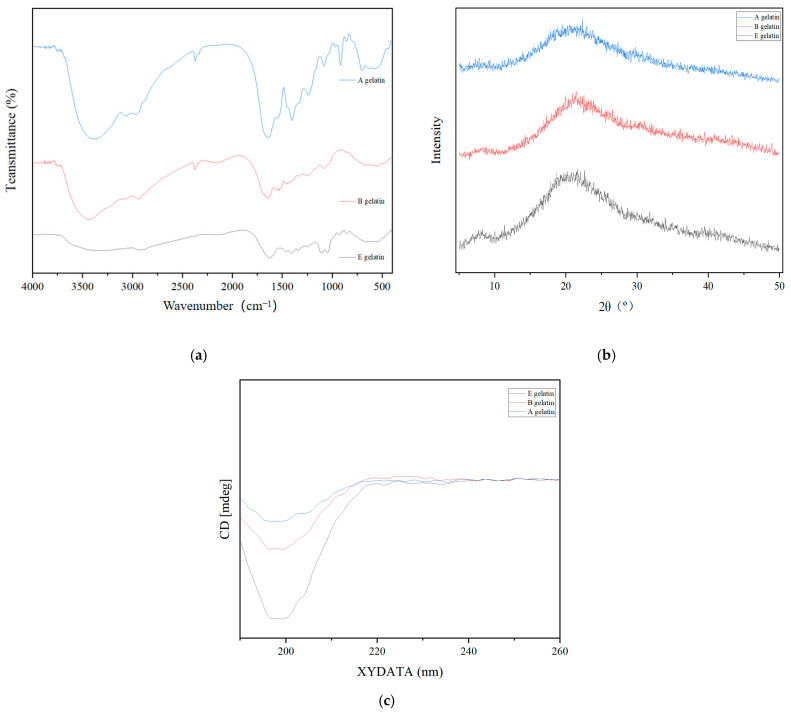
FTIR spectra: (**a**) XRD patterns and (**b**) CD spectra (**c**) of A gelatin, B gelatin, and E gelatin.

SDS-PAGE analysis of gelatin extracted from Yanbian cattle bones using different methods—acidic (A gelatin), alkaline (B gelatin), and enzymatic (E gelatin)—revealed notable differences in the preservation of collagen protein subunits (Figure 3a). All three extraction methods displayed distinct bands at around 140 kDa, corresponding to the α1 and α2 chains, characteristic of collagen proteins. This result is consistent with previous studies on gelatin [14,41,42,43]. However, the intensity and clarity of these bands varied, reflecting the degree of preservation or degradation of the collagen structure during extraction. For acid-extracted gelatin (A), the α1 and α2 bands were relatively weak, indicating that the acidic environment caused significant structural breakdown, resulting in partial hydrolysis of collagen. This finding correlates with the prior FTIR analysis, which suggested that acid extraction reduces the integrity of the protein structure, resulting in a lower molecular weight and potentially reduced gel strength and viscosity. Hydrochloric acid protonates the amino and carboxyl functional groups in collagen, disrupting the hydrogen bonds and other non-covalent interactions between collagen molecules, leading to their unraveling and degradation. As the structure is damaged, the collagen molecules become shorter and irregular, resulting in a significant decrease in molecular weight. The smaller molecular weight prevents the gelatin from forming a strong gel network upon cooling, which causes a reduction in gel strength and viscosity [44]. In contrast, alkaline-extracted gelatin (B) exhibits clearer and stronger bands at α1 and α2, suggesting better retention of collagen subunits. However, the use of a strong alkali, such as sodium hydroxide, causes some degradation, as evidenced by slightly diffuse bands. This intermediate retention of the collagen structure positions alkali-extracted gelatin between acidic and enzymatic methods in terms of gel strength and functional properties, making it suitable for general food additive applications. Notably, enzymatic extraction (E) shows the most prominent and well-defined α1 and α2 bands, indicating minimal structural damage during the extraction process. Selective hydrolysis by papain enabled efficient extraction while preserving high-molecular-weight components. These results align with previous FTIR findings, which demonstrated superior retention of the protein’s secondary structure and functional properties (Figure 4a). Consequently, enzymatically extracted gelatin offers a higher gel strength and viscosity, making it ideal for high-value applications in pharmaceuticals and premium food formulations [45]. This underscores the significance of the molecular weight distribution, structure, and subunit composition of gelatin (including α chains and low-molecular-weight protein fragments) in determining the functional and physical properties of gelatin.

#### 2.4.2. GPC

Gel Permeation Chromatography (GPC) analysis of gelatin extracted from Yanbian cattle bones using different methods—acid extraction (A gelatin), alkaline extraction (B gelatin), and enzymatic extraction (E gelatin)—demonstrated significant differences in molecular weight distribution among the samples (Figure 3b,c). The GPC chromatogram revealed that gelatin A (acid-extracted) exhibited a major peak at approximately 26 min, indicating a lower molecular weight due to extensive hydrolysis caused by acidic conditions, which generated a higher proportion of small molecular fragments. In contrast, gelatin B (alkaline extract) showed a broader distribution, with peaks between 24 and 28 min, reflecting the presence of both high- and low-molecular-weight components. This suggests that, while alkaline treatment partially degrades collagen structure, it retains a moderate level of molecular integrity compared to the acid method. Notably, gelatin E (enzymatically extracted) showed a prominent peak at an earlier retention time (15–20 min), indicative of higher-molecular-weight fractions. Selective hydrolysis by papain preserved larger collagen chains, resulting in shorter retention times in the chromatogram. Papain selectively hydrolyzes collagen through its specific and mild enzymatic action, and the mechanism by which it protects larger collagen chains is primarily reflected in its selective hydrolysis characteristics. Papain is a cysteine protease, and its mechanism involves a catalytic triad consisting of cysteine, histidine, and asparagine residues. The enzyme selectively targets peptide bonds that are more susceptible to hydrolysis, while preserving those bonds that are crucial for the structural integrity of the collagen molecule. The protease cleaves peptide bonds at specific sites within the collagen molecule, thereby removing non-collagenous impurities, while retaining key structural components of collagen, particularly the larger collagen chains [16]. The molecular weight distribution chart further supports these observations. For gelatin A, 40% of the content was in the 10–100 kDa range, 32% was below 10 kDa, and only 13% exceeded 300 kDa, indicating a high proportion of low-molecular-weight fragments. Gelatin B displayed a relatively balanced distribution, with 34% in the <10 kDa range, 38% in the 10–100 kDa range, and smaller fractions in the 100–300 kDa (14%) and >300 kDa (13%) ranges, suggesting moderate degradation and a diverse molecular weight profile suitable for general food additive applications. In contrast, gelatin E showed a significant shift toward higher molecular weights, with 39% of its content exceeding 300 kDa and only 3% below 10 kDa, reflecting minimal structural breakdown during enzymatic extraction.

### 2.5. Functional Groups of Gelatin

Table 3 and Figure 4a present the FTIR spectra, wavenumber ranges, and assignments of the gelatin extracted from Yanbian cattle bone. The Amide A band for the three types of gelatin (A gelatin, B gelatin, and E gelatin) exhibited wavenumber ranges of 3348.42–3435.22 cm^−1^. Gelatin A and B exhibited higher wavenumbers, attributed to hydrolysis by hydrochloric acid and sodium hydroxide, which altered the hydrogen-bonding environment of the protein [46]. This process weakened hydrogen bonding and increased the freedom of N–H stretching vibrations [47]. Similar results were reported by Ahmad et al. when extracting gelatin using different enzymes [48,49]. The Amide B band appeared within 2931.8–3064.89 cm^−1^, with gelatin A showing the highest wavenumber (3064.89 cm^−1^). This increase was attributed to acid hydrolysis, which reduces molecular cross-linking and enhances molecular vibrational freedom [50]. The Amide I band, primarily arising from C=O stretching vibrations, serves as a critical fingerprint region for protein secondary structure [51]. The results indicated that all three types of gelatin retained a certain degree of protein secondary structure. This result ties well with previous studies by Ahmad et al., Samatra et al., and Ma et al. who found a similar wavenumber for gelatin pre-treated with different types of enzymes [14,27,48,52]. The wavenumber is generally indicative of the hydrogen-bonding strength involving the carbonyl group (C=O), with higher wavenumbers indicating weaker hydrogen bonds [53]. Gelatin E displayed the lowest wavenumber (1629.85 cm^−1^), suggesting that papain hydrolysis led to stronger hydrogen bonding involving carbonyl groups compared to the other two hydrolysis methods. The amide II band, mainly consisting of N–H bending and C–N stretching vibrations, is influenced by hydrogen bonding and the degree of protein folding [54]. Gelatin A showed the lowest wavenumber (1462.04 cm^−1^), indicating that papain hydrolysis has a lesser impact on molecular cross-linking and hydrogen bonding compared to hydrolysis by hydrochloric acid or sodium hydroxide. The amide III band, generated by a combination of C–N stretching and N–H bending vibrations, is another important region for studying protein secondary structures [55]. Gelatin E exhibited the highest wavenumber (1247.94 cm^−1^), indicating that enzymatic hydrolysis has the least effect on the stability of C–N bonds. Meanwhile, a lower amplitude indicates a significant shift to a disordered coiled structure due to molecular disorder [48].

Overall, among the three types of gelatins, E gelatin had a more compact molecular structure, lower vibration wavenumbers, and the most intact protein secondary structure. Gelatin B exhibited moderate structural damage with intermediate wavenumbers, while gelatin A showed the greatest disruption of hydrogen bonding and secondary structures, resulting in higher wavenumbers for most vibration bands and increased freedom from molecular vibrations.

### 2.6. XRD Analysis

XRD analysis is commonly used to analyze the crystallinity and triple-helical structure of gelatin [56]. Based on the XRD analysis of gelatin extracted from Yanbian cattle bones using acid (A gelatin), alkaline (B gelatin), and enzymatic (E gelatin) methods, distinct differences in crystallinity and structural characteristics were observed (Figure 4b). All three types of gelatin exhibited broad diffraction peaks, reflecting primarily an amorphous structure with some degree of crystallinity. Gelatin, a denatured form of collagen, generally lacks a highly ordered structure but retains minor crystalline regions due to residual sequences from the native triple helix [57]. The XRD pattern of A gelatin (acid-extracted) shows a broad, low-intensity peak centered around 21° (2θ), suggesting significant disruption of the collagen structure by acid treatment. This led to a more amorphous gelatin with minimal crystalline regions, a result of extensive hydrolysis under acidic conditions that degraded ordered structures. In contrast, B gelatin (alkaline-extracted) displayed a moderately sharper and higher-intensity peak around 21° (2θ), indicating partial retention of the crystalline regions of the original collagen. Alkaline extraction caused less aggressive degradation compared to acid treatment, preserving a moderate degree of structural order. Notably, E gelatin (enzymatically extracted) showed the highest peak intensity at around 20° (2θ), indicating that enzymatic extraction best preserved the ordered regions within the gelatin structure. The sharper and more intense peaks reflect a higher degree of crystallinity, likely due to selective hydrolysis by enzymes such as papain, which minimizes structural disruption while effectively extracting gelatin [58].

### 2.7. Amino Acid Composition of Gelatin

The concentration of amino acids in gelatin varies depending on the source and extraction method used [59,60]. Table 4 presents the amino acid composition of Yanbian cattle bone gelatin prepared using acidic (A gelatin), alkaline (B gelatin), and enzymatic (E gelatin) methods, each showing distinct variations. Hydrochloric acid extraction under acidic conditions tends to hydrolyze and degrade collagen chains. Under acidic conditions, some essential amino acids, such as glycine, glutamic acid, and leucine, are subject to varying degrees of damage and loss. Glutamic acid and leucine are crucial for collagen properties; however, the strong hydrolytic ability of the acid extraction method disrupts the triple-helix structure of collagen, resulting in lower levels of these amino acids compared to B gelatin and E gelatin. Furthermore, basic amino acids, such as lysine, are prone to partial degradation under acidic conditions, leading to reduced lysine content. Consequently, gelatin extracted using the acid method exhibited lower amounts of glycine, glutamic acid, and lysine, as well as a lower overall amino acid composition. In contrast, extraction using sodium hydroxide (the alkaline method) retains certain amino acids due to the higher pH, although it still leads to collagen degradation. Amino acids such as glycine, glutamic acid, and leucine demonstrate higher stability in alkaline environments. This suggests that, compared to gelatin A, gelatin B retains more of its original collagen structure. Studies have shown that alkaline treatment helps remove non-collagenous substances, resulting in purer collagen. When properly controlled, this method preserves more of the native collagen structure [61].

Enzymatic extraction (using papain) causes the least damage to collagen due to its high specificity and mild reaction conditions. This method effectively preserves the natural structure of collagen, resulting in higher levels of key amino acids such as glycine, glutamic acid, lysine, proline, and hydroxyproline. These amino acids are essential for maintaining the triple-helical structure of gelatin, as well as its physical and chemical properties, such as gel strength and thermal stability. Moreover, enzymatic extraction conducted at lower temperatures and under neutral to mildly acidic conditions minimizes the risk of amino acid degradation, particularly for basic amino acids such as lysine. As a result, gelatin extracted using the enzymatic method exhibited a more complete amino acid composition and optimal physical and functional properties [15].

### 2.8. Circular Dichroism Spectrum of Gelatin

CD analysis of gelatin extracted from Yanbian cattle bones using acidic (A gelatin), alkaline (B gelatin), and enzymatic (E gelatin) methods revealed significant differences in the secondary structure of gelatin (Figure 4c and Table 5). E gelatin exhibited a relatively more ordered structure, characterized by prominent peaks around 210 nm, indicating a higher content of α-helix and β-sheet [62,63]. The secondary structure analysis showed that E gelatin contained 14.4% α-helix and 27.1% β-sheet, suggesting that enzymatic extraction preserved the collagen triple helix and retained more ordered structures. B gelatin, Similar results were reported by Ma et al. when extracting gelatin using different enzymes [14], on the other hand, showed a more balanced secondary structure with a significant proportion of β-turn (35.5%) and unordered coil (35.6%), while its α-helix content was 8.7% and β-sheet content was 20.2%. The CD spectrum for B gelatin exhibited a more gradual curve, indicating a less ordered structure compared to E gelatin but still more stable than A gelatin. A gelatin exhibited a more disordered structure, as indicated by the strong absorption peaks and higher content of unordered coil (44.9%) and β-turn (28.2%). The secondary structure analysis revealed that A gelatin had only 1.4% α-helix and 25.5% β-sheet, with a predominance of disordered regions likely due to the harsh conditions of acid extraction. These findings suggest that enzymatic extraction (E gelatin) better preserves the collagen triple helix, resulting in more ordered structures, while acid extraction (A gelatin) leads to excessive degradation and a greater proportion of disordered structures.

## 3. Conclusions

This study comprehensively evaluated the extraction efficiency and physicochemical properties of gelatin obtained from Yanbian cattle bones using acid, alkaline, and enzymatic methods. The results demonstrated that enzymatic hydrolysis with papain produced the highest yield and preserved collagen structure, as evidenced by superior hydroxyproline content, gel strength, viscosity, and molecular weight distribution. Compared to acid and alkaline methods, enzymatic extraction minimized structural damage, retained functional groups, and maintained higher crystallinity, as confirmed by FTIR and XRD analyses. These findings suggest that papain-assisted enzymatic hydrolysis is an effective method for preparing bone gelatin from Yanbian cattle. Yanbian cattle bone gelatin shows promise as a viable halal gelatin alternative to traditional mammalian gelatin, with broad application prospects in fields such as food and pharmaceuticals.

## 4. Material and Methods

### 4.1. Materials

Yanbian cattle bones were purchased from the Yanji market and stored at −20 °C before use. Pepsin (15,600 U/g), papain (800 U/mg), ficin (40,000 U/g), ginger protease (400 U/mg), compound protease (120 U/mg), bromelain (50,000 U/g), pancreatin (100 U/mg), and trypsin (250 U/g) were purchased from Shanghai Yuanye Biotechnology Co., Ltd. (Shanghai, China). Analytical-grade chemicals were obtained from Tianjin Kemiou Chemical Reagent Co., Ltd. (Tianjin, China).

### 4.2. Pretreatment

Yanbian cattle bone powder was prepared based on the method described by Cao et al. [20], with minor modifications. The Yanbian cattle bone was thoroughly washed with distilled water and soaked in distilled water for 3 h to remove blood. The bone was then dried in an oven at 40 °C for 24 h. Subsequently, the dried bone was pulverized using a high-speed grinder and sieved through a 60-mesh sieve to obtain Yanbian cattle bone powder. The bone powder was mixed with 0.25 M EDTA (1:10, *v*/*v*) at 20 °C to remove calcium salts. The EDTA solution was replaced every 12 h for three consecutive days. After the demineralization process, the slurry was centrifuged at 12,000 rpm for 10 min at 4 °C to remove the supernatant. The residue was concentrated using a rotary evaporator, (Buchi, Flawil, St. Gallen, Switzerland) followed by freeze-drying to obtain demineralized Yanbian cattle bone powder. The resulting powder was stored at −80 °C for further use.

### 4.3. Gelatin Extraction

#### 4.3.1. Enzymatic Extraction and Enzyme Selection

A total of 4.00 g of pre-treated Yanbian cattle bone powder was weighed, and enzymatic hydrolysis was performed under the optimal conditions (temperature and pH) for different enzymes. The conditions for each enzyme were as follows: pepsin (37 °C, pH 3), papain (60 °C, pH 6.5), ficin (37 °C, pH 10), ginger protease (60 °C, pH 6.5), compound protease (50 °C, pH 7.5), bromelain (60 °C, pH 7), pancreatin (37 °C, pH 7.5), and trypsin (37 °C, pH 8). The enzyme dosage was 140 U/g, with a solid-to-liquid ratio of 1:7 (*w*/*v*). After 3 h of enzymatic hydrolysis, the enzymes were inactivated by boiling in a water bath at 100 °C for 10 min. The resulting mixture was then extracted with deionized water at 60 °C for 5 h. After extraction, based on the method of Ma et al., the mixture was centrifuged at 12,000 rpm for 10 min, and the supernatant was collected. The supernatant was filtered through Whatman No. 4 filter paper to obtain a gelatin solution. The solution was subsequently frozen at −80 °C for 24 h and freeze-dried to obtain enzymatically treated bovine bone gelatin samples [14]. The most suitable enzyme was determined by comparing the gelatin yield from different treatments.

#### 4.3.2. Acid Extraction and Determination of HCl Concentration

Based on the method of Cao et al. and Samatra et al. [13,20], with slight modifications, we aimed to maximize gelatin yield while avoiding excessive degradation of bovine bone collagen due to hydrochloric acid treatment; 4.00 g of pre-treated bovine bone powder was weighed and added to 1:7 (*w*/*v*) mixtures of hydrochloric acid solutions with concentrations of 0.025, 0.05, 0.075, 0.1, 0.125, and 0.15 mol/L. The mixtures were gently stirred at 4 °C for 24 h, with the solution replaced every 6 h. After treatment, the bovine bone residue was washed repeatedly with distilled water until the pH of the wash water became neutral. The washed bone powder was air-dried and weighed. The treated samples were mixed with deionized water at a 1:7 solid-to-liquid ratio (*w*/*v*) and extracted in a water bath at 60 °C for 5 h. After extraction, the mixture was centrifuged at 12,000 rpm for 10 min, and the supernatant was collected. The supernatant was filtered through Whatman No. 4 filter paper (Whatman, Maidstone, Kent, UK) to obtain a gelatin solution. The solution was then frozen at −80 °C for 24 h and freeze-dried to obtain acid-treated gelatin samples. The most suitable HCl concentration was determined by comparing the gelatin yield from different treatments.

#### 4.3.3. Alkaline Extraction and Determination of NaOH Concentration

Slight modifications were made based on the experimental method of Ahmad et al. [48]. A total of 4.00 g of pre-treated bovine bone powder was weighed and added to 1:7 (*w*/*v*) mixtures of sodium hydroxide solutions with concentrations of 0.025, 0.05, 0.075, 0.1, 0.125, and 0.15 mol/L. The mixtures were gently stirred at 4 °C for 24 h, with the solution replaced every 6 h. After treatment, the bovine bone residue was washed repeatedly with distilled water until the pH of the wash water became neutral. The washed bone powder was air-dried and weighed. The treated samples were mixed with deionized water at a 1:7 solid-to-liquid ratio (*w*/*v*) and extracted in a water bath at 60 °C for 5 h. After extraction, the mixture was centrifuged at 12,000 rpm for 10 min, and the supernatant was collected. The supernatant was filtered through Whatman No. 4 filter paper to obtain a gelatin solution. The solution was then frozen at −80 °C for 24 h and freeze-dried to obtain alkaline-treated gelatin samples. The most suitable NaOH concentration was determined by comparing the gelatin yield from different treatments.

### 4.4. Extraction Yield of Yanbian Cattle Bone Gelatin

The yield of gelatin was calculated using the following Equation (1):

(1) $$\mathrm{Yield}\;\mathrm{of}\;\mathrm{Yanbian}\;\mathrm{cattle}\;\mathrm{bone}\;\mathrm{gelatin}\;(\%)=\frac{W_{g}}{W_{b}}\times 100,$$

where W_g_ is the weight of dry gelatin and W_b_ is the wet weight of Yanbian cattle bone.

### 4.5. Characterization of Yanbian Cattle Bone Gelatin

In this experiment, the gelatin samples with the highest extraction yields from the three preparation methods were selected for further analysis. They were named as A gelatin, B gelatin, and E gelatin. The quality of bone gelatin was evaluated based on key indicators, including chemical properties, hydroxyproline content, gel strength, and viscosity. Additionally, supplementary parameters such as amino acid composition, molecular weight distribution and structure, rheological properties, crystal structure, and protein structure were also analyzed.

#### 4.5.1. Chemical Properties

The moisture, ash, and crude protein contents were determined following the methods outlined in (AOAC), 2000 [64]. Each measurement was conducted in triplicate.

#### 4.5.2. Hydroxyproline Content

Hydroxyproline content was determined as described by Ristaniemi et al. [38]. A 20 mg sample of freeze-dried gelatin was weighed and mixed with 3 mL of hydrochloric acid (6 mol/L). The mixture was hydrolyzed at 130 °C for 4 h. After cooling, the solution was transferred to a small beaker, and one drop of methyl red reagent was added. Subsequently, 1 mL of citrate buffer and 1 mL of chloramine-T solution were added, and the mixture was allowed to react at room temperature for 10 min. Next, 1 mL of perchloric acid (3.5 mol/L) was added, and the reaction was continued at room temperature for another 10 min. Afterward, 1 mL of color reagent was introduced, and the reaction was incubated in a water bath at 65 °C for 20 min. The mixture was then cooled to room temperature, and the absorbance was measured at 558 nm. The hydroxyproline content was quantified using a standard curve derived from hydroxyproline as a reference.

#### 4.5.3. Gel Strength

The gelatin solution, consisting of 120 mL at a concentration of 6.67% (*w*/*v*), was transferred into a Bloom jar and matured at 10 °C for 17 h. Gel strength was assessed using the TA. XT Plus Texture Analyzer (Stable Micro Systems, England, UK) equipped with a flat-ended cylindrical probe with a diameter of 4.12 mm. The probe was set to descend at a constant rate of 0.5 mm/s and was allowed to penetrate the gel to a depth of 4 mm. The test was performed once, and the peak force (measured in g) encountered during the compression was recorded as the gel strength. The compression test was carried out at room temperature (approximately 20 °C) [19].

#### 4.5.4. Viscosity

Gelatin (1.34 g) was dissolved in distilled water (20 mL) to prepare a 6.67% (*w*/*v*) gelatin solution, which was then heated to 60 °C. The viscosity of the solution was measured using a RheolabQC viscometer (Anton Paar, Graz, Austria). Triplicate measurements were performed to ensure accuracy, and the mean value was calculated [27].

#### 4.5.5. Measurement of ζ -Potential and pH

The pH of Yanbian cattle gelatin was measured according to the method described by Ahmad et al. [65]. A 1% (*w*/*v*) gelatin solution was prepared by dissolving 0.2 g of gelatin in 20 mL of distilled water and cooling it at 25 °C. The pH meter (Thermo Fisher Scientific, Waltham, MA, USA) was calibrated with buffer solutions of pH 4.0 and 7.0, and measurements were repeated three times.

The ζ-potential analyzer (Zeta PALS, Malvern, UK) was used to measure the ζ-potential at room temperature (25 °C). Gelatin solution (0.5 mg/mL) dissolved in deionized water was adjusted to different pH levels (2, 3, 4, 5, 6, 7, 8, 9, 10, 11) using 0.1 M hydrochloric acid or 0.1 M sodium hydroxide. The isoelectric point (pI) was determined as the pH at which the ζ-potential reached zero.

#### 4.5.6. Rheological Properties

The rheological behavior of the 6.67% (*w*/*v*) gelatin solution was investigated using an AR G2 rheometer (TA Instruments Inc., New Castle, DE, USA) equipped with a parallel-plate geometry with a diameter of 40 mm. Temperature sweeps were conducted from 5 °C to 60 °C and back to 5 °C at a rate of 2 °C/min, with a strain of 1% and a frequency of 1 Hz. The storage modulus (G′) and loss modulus (G′) were recorded during the temperature scans. All measurements were performed within the linear viscoelastic region of the rheometer, and each experiment was repeated at least three times.

#### 4.5.7. Electrophoresis Analysis of Gelatin (SDS-PAGE)

Electrophoretic analysis was employed to evaluate the molecular weight distribution of the gelatin samples, as previously reported [66]. For the SDS-PAGE gel electrophoresis (6% stacking gel, 8% separating gel), gelatin samples (0.1 mg/mL) were mixed with 5× sample loading buffer in a 1:4 ratio, followed by heating in a boiling water bath for 5 min. A 10 μL aliquot of the sample was then loaded onto the gel. Electrophoresis was initially performed at 80 V, and once the bands entered the separating gel, the voltage was increased to 120 V until the electrophoresis was complete. After electrophoresis, the polyacrylamide gel was carefully removed and stained with Coomassie Brilliant Blue R-250 solution at room temperature for 30 min. The gel was then washed with distilled water to remove excess stain and destained with a decolorizing solution until the bands were clearly visible. Finally, the destained gel was photographed using a gel imaging system.

#### 4.5.8. Molecular Weight Distribution (GPC)

The relative molecular weight distribution of gelatin proteins was determined using an Agilent 1260 High-Performance Liquid Chromatography (HPLC) system (Agilent Technologies, Santa Clara, CA, USA) coupled with three Waters Ultrahydrogel columns (TM120, TM250, and TM500 (Waters Corporation, Milford, MA, USA); dimensions: 7.8 mm × 300 mm). The experimental conditions included a flow rate of 1 mL/min, column and detector temperatures of 40 °C, and detection with a G1362A differential refractive index detector. The mobile phase consisted of a 0.1 M aqueous solution of NaNO_3_.

For sample preparation, 0.020 g of gelatin was dissolved in 4 mL of the mobile phase using ultrasonic dissolution and filtered through a 0.45 μm filter membrane. A 40 μL sample was injected into the system, and the analysis runtime was 35 min. The relative molecular weight distribution of the gelatin proteins was determined by analyzing the obtained chromatograms.

#### 4.5.9. Fourier Transform Infrared Spectroscopy (FT-IR)

The Fourier transform infrared (FTIR) spectra of gelatin were obtained using an FTIR spectrometer (Shimadzu, IRTRACER-100, Kyoto, Japan). Dried gelatin powder was analyzed at 25 °C, and transmittance (%) data were recorded from 400 cm^−1^ to 4000 cm^−1^. The results were plotted as a function of the wavenumber (cm^−1^).

#### 4.5.10. X-Ray Diffraction (XRD)

XRD patterns of the gelatin samples were acquired using an X-ray diffractometer (Bruker AXS, Rheinfelden, Germany). Gelatin powder was evenly dispersed on a sample plate and scanned over a 2θ range from 5° to 50° in 0.05° increments. Cu Kα radiation was employed as the X-ray source, operating at 30 kV and 10 mA. The resulting XRD data were exported and visualized using OriginPro2021 graph-plotting software.

#### 4.5.11. Amino Acid Analysis

Gelatin samples were hydrolyzed with 6 mol/L HCl in a hydrolysis tube and processed as previously described. After purging with nitrogen for 1 min to exclude air, the samples were heated at 110 °C for 24 h. The hydrolysates were filtered through a 0.45 μm filter membrane, and the filtrates were analyzed using an LC-MS/MS system (Shimadzu, LCMS-8050, Kyoto, Japan) equipped with a C18 chromatographic column (Discovery HS F5-3, 15 cm × 2.1 mm, 3 μm, Supelco, Bellefonte, Pennsylvania, USA). The column temperature was maintained at 35 °C, and the flow rate was 0.6 mL/min. Data were processed using LabSolutions Ver. 5.99 SP3 software and LabSolutions Insight™ Ver. 3.5 (Shimadzu Corporation, Kyoto, Japan) [67].

#### 4.5.12. Circular Dichroism (CD) Spectroscopy

The circular dichroism (CD) spectrum of gelatin was measured at 25 °C using a J-815 CD spectropolarimeter (Jasco, Tokyo, Japan). Approximately 100 mL of gelatin solution (0.5 mg/mL, dissolved in deionized water) was pipetted into a cuvette and scanned from 190 to 260 nm at a scanning speed of 50 nm/min.

#### 4.5.13. Statistical Analysis

All experiments were conducted in triplicate, and the results are expressed as the mean ± standard deviation (SD). Statistical analysis of the data was performed using analysis of variance (ANOVA). To further analyze the test data, a multiple-range test was conducted using SPSS Statistics 23.0. Graphical representation of the data was achieved using Origin 2021 software. The means were compared using Duncan’s test at a significance level of *p* < 0.05.

## Figures and Tables

**Table 1:** Hydroxyproline and chemical properties of Yanbian cattle bone gelatin.

Type of Gelatin	Hydroxyproline Content (g/100 g)	Moisture (%)	Ash (%)	Crude Protein (%)
A gelatin	11.51 ± 0.20 ^c^	7.67 ± 0.29 ^a^	0.94 ± 0.07 ^a^	77.57 ± 0.46 ^c^
B gelatin	13.48 ± 0.35 ^b^	7.47 ± 0.26 ^a^	1.00 ± 0.06 ^a^	84.16 ± 0.42 ^b^
E gelatin	19.13 ± 0.13 ^a^	7.40 ± 0.11 ^a^	1.00 ± 0.09 ^a^	87.54 ± 0.40 ^a^

Results are presented as mean ± SD (*n* = 3). Statistical analyses were performed on samples with the same properties. Different superscript letters (a–c) within the same column indicate statistically significant differences, as determined by one-way ANOVA followed by Duncan’s post hoc test (*p* < 0.05).

**Table 2:** Gel strength, viscosity, pH, and isoelectric point of Yanbian cattle bone gelatin.

Type of Gelatin	Gel Strength (g)	Viscosity (cP)	pH	Isoelectric Point
A gelatin	197.33 ± 13.58 ^c^	484.33 ± 5.13 ^b^	5.31 ± 0.01 ^c^	7.44 ± 0.22 ^b^
B gelatin	207.67 ± 11.59 ^b^	482.67 ± 6.81 ^b^	7.41 ± 0.01 ^a^	5.42 ± 0.06 ^c^
E gelatin	259.00 ± 10.54 ^a^	521.67 ± 7.37 ^a^	6.44 ± 0.01 ^b^	8.5 ± 0.14 ^a^

Results are presented as mean ± SD (*n* = 3). Statistical analyses were performed on samples with the same properties. Different superscript letters (a–c) within the same column indicate statistically significant differences, as determined by one-way ANOVA followed by Duncan’s post hoc test (*p* < 0.05).

**Table 3:** FTIR spectra wavenumber and assignments of Yanbian cattle bone gelatin.

Type of Gelatin	Wavenumber (cm^−1^)				
	Amide A	Amide B	Amide I	Amide II	Amide III
A gelatin	3390.86	3064.89	1643.35	1550.77	1238.30
B gelatin	3435.22	2935.66	1641.42	1529.55	1242.16
E gelatin	3348.42	2931.80	1629.85	1462.04	1247.94
Assignment	Corresponds to the N–H stretching vibration	Corresponds to another mode of N–H stretching vibration	Primarily arises from the C=O stretching vibration	Mainly composed of N–H bending vibration and C–N stretching vibration	Results from a combination of C–N stretching and N–H bending vibrations

**Table 4:** Amino acid compositions (residues per 100%) of A gelatin, B gelatin, and E gelatin.

Amino Acid	A Gelatin	B Gelatin	E Gelatin
Asp	3.06 ± 0.37	5.01 ± 0.41	5.06 ± 0.33
Thr	1.03 ± 0.58	1.90 ± 0.83	1.95 ± 0.78
Ser	2.46 ± 0.26	3.43 ± 0.11	3.36 ± 0.16
Glu	6.23 ± 0.27	8.65 ± 0.22	8.27 ± 0.17
Gly	33.25 ± 0.48	37.40 ± 0.51	36.55 ± 0.41
Ala	13.19 ± 0.35	14.08 ± 0.21	14.09 ± 0.28
Cys	0.00 ± 0.00	0.00 ± 0.00	0.00 ± 0.00
Val	1.96 ± 0.80	2.46 ± 0.74	2.42 ± 0.79
Met	0.29 ± 0.55	0.75 ± 0.32	0.54 ± 0.27
Ile	1.06 ± 0.58	1.37 ± 0.62	1.36 ± 0.63
Leu	2.17 ± 0.21	2.97 ± 0.12	3.01 ± 0.11
Tyr	0.14 ± 0.05	0.29 ± 0.42	0.31 ± 0.35
Phe	1.37 ± 0.34	1.49 ± 0.51	1.54 ± 0.44
Lys	2.46 ± 0.15	3.03 ± 0.11	3.16 ± 0.25
NH3	3.15 ± 0.08	3.83 ± 0.22	3.78 ± 0.18
His	0.47 ± 0.21	0.59 ± 0.31	0.61 ± 0.23
Arg	4.83 ± 0.18	5.54 ± 0.25	5.52 ± 0.22
Imino Acid (Pro + Hyp)	12.26 ± 0.11	16.44 ± 0.12	22.31 ± 0.07

**Table 5:** Content of the secondary structure in three varieties of Gelatin (%).

Type of Gelatin	α-Helix	β-Sheet	β-Turn	Unordered Coil
A gelatin	1.4 ± 0.2	25.5 ± 0.1	28.2 ± 0.1	44.9 ± 0.2
B gelatin	8.7 ± 0.2	20.2 ± 0.3	35.5 ± 0.3	35.6 ± 0.1
E gelatin	14.4 ± 0.1	27.1 ± 0.2	35.2 ± 0.2	23.3 ± 0.1

## Data Availability

The raw data supporting the conclusions of this article will be made available by the authors on request.
